# Protective Effect of Diphlorethohydroxycarmalol against Ultraviolet B Radiation-Induced DNA Damage by Inducing the Nucleotide Excision Repair System in HaCaT Human Keratinocytes

**DOI:** 10.3390/md13095629

**Published:** 2015-09-02

**Authors:** Mei Jing Piao, Susara Ruwan Kumara Madduma Hewage, Xia Han, Kyoung Ah Kang, Hee Kyoung Kang, Nam Ho Lee, Jin Won Hyun

**Affiliations:** 1 School of Medicine and Institute for Nuclear Science and Technology, Jeju National University, Jeju 63243, Korea; E-Mails: meijing0219@hotmail.com (M.J.P.); susaramh@gmail.com (S.R.K.M.H.); hanx55@hotmail.com (X.H.); legna07@naver.com (K.A.K.); pharmkhk@jejunu.ac.kr (H.K.K.); 2 Department of Chemistry, College of Natural Sciences, Jeju National University, Jeju 63243, Korea; E-Mail: namho@jejunu.ac.kr

**Keywords:** diphlorethohydroxycarmalol, ultraviolet B, cyclobutane pyrimidine dimmers, xeroderma pigmentosum complementation group C, excision repair cross-complementing 1

## Abstract

We investigated the protective properties of diphlorethohydroxycarmalol (DPHC), a phlorotannin, against ultraviolet B (UVB) radiation-induced cyclobutane pyrimidine dimers (CPDs) in HaCaT human keratinocytes. The nucleotide excision repair (NER) system is the pathway by which cells identify and repair bulky, helix-distorting DNA lesions such as ultraviolet (UV) radiation-induced CPDs and 6-4 photoproducts. CPDs levels were elevated in UVB-exposed cells; however, this increase was reduced by DPHC. Expression levels of xeroderma pigmentosum complementation group C (XPC) and excision repair cross-complementing 1 (ERCC1), which are essential components of the NER pathway, were induced in DPHC-treated cells. Expression of XPC and ERCC1 were reduced following UVB exposure, whereas DPHC treatment partially restored the levels of both proteins. DPHC also increased expression of transcription factor specificity protein 1 (SP1) and sirtuin 1, an up-regulator of XPC, in UVB-exposed cells. DPHC restored binding of the SP1 to the XPC promoter, which is reduced in UVB-exposed cells. These results indicate that DPHC can protect cells against UVB-induced DNA damage by inducing the NER system.

## 1. Introduction

Ultraviolet B (UVB) radiation (280–320 nm), a component of sunlight, can cause severe damage to skin cells and even induces skin cancer [1]. UVB radiation is one of the most important causes of three major classes of DNA lesions: cyclobutane pyrimidine dimers (CPDs), pyrimidine 6-4 pyrimidone photoproducts (6-4PPs), and their Dewar isomers [2]. CPDs, the most prominent type, is formed approximately ten times more frequently than the corresponding 6-4PPs, making CPDs the major photoproduct of UVB radiation [3].

Various DNA repair pathways respond to DNA damage induced by oxidative stress and exposure to environmental factors such as UV radiation. DNA repair plays a major role in maintaining genomic stability via multiple pathways. The canonical nucleotide excision repair (NER) pathway removes helix-distorting nucleotide lesions, including UV light-induced CPDs. Mutations in genes of the NER pathway, including xeroderma pigmentosum group C (XPC) and excision repair cross complementation group 1 (ERCC1), are associated with elevated risk for various cancers. The XPC protein is an important DNA damage-recognition protein involved in global genome DNA repair (GGR), a sub-pathway of NER [4,5]. XPC recognizes a variety of bulky DNA lesions, including UV-induced CPDs [6]. XPF and ERCC1 exist as a heterodimer. Together with XPF, ERCC1 excises a short oligonucleotide encompassing the UV-induced lesion, and the resulting gap is filled by replicative DNA polymerases that use the complementary strand as a template [7,8].

*Ishige okamurae*, an edible brown alga, is abundant along the coast of Jeju Island (Korea). Diphlorethohydroxycarmalol (DPHC), a phlorotannin isolated from *Ishige okamurae*, has antioxidant [9,10,11], antiviral [12], hypoglycemic [13], and anti-melanogenesis properties [14]; in addition, it increases the effects of prostaglandin E_2_ [15]. In particular, DPHC also exerts protective effects against UVB and gamma radiation [11,14,16]. Previously, we reported that DPHC protects epidermal cells against damage caused by UVB light [11], although at that time the mechanism was unclear. The purpose of this study was to elucidate the cytoprotective mechanisms underlying DPHC-mediated protection against UVB-induced DNA damage, especially in relation to the NER repair system.

## 2. Results

### 2.1. Effect of DPHC against UVB-Induced CPDs Formation

**Figure 1:**
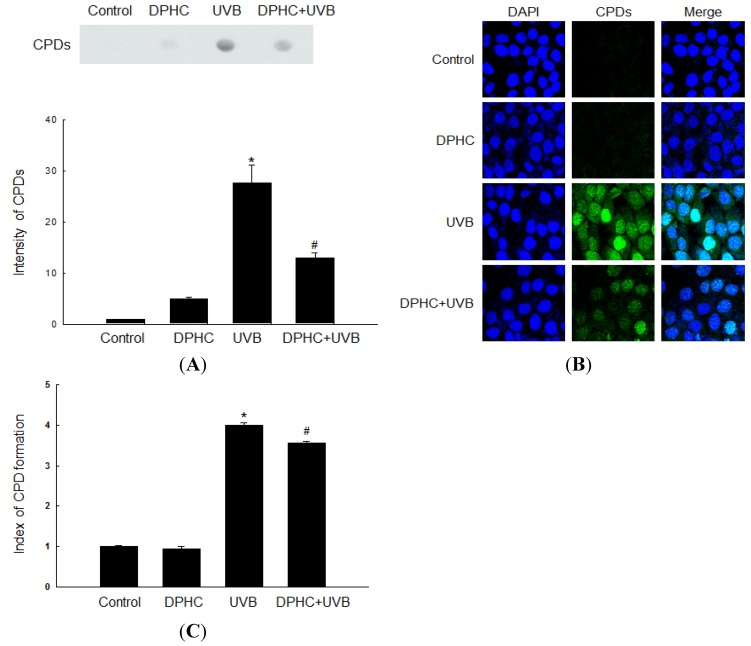
DPHC decreases UVB-induced cyclobutane pyrimidine dimers (CPDs) formation in HaCaT cells. Cells were incubated with 20 μM DPHC for 1 h and irradiated with UVB at 300 J/m^2^. Twenty-four hours after UVB exposure, (**A**) genomic DNA was extracted and analyzed by dot blot using an anti-CPD antibody; (**B**) Immune-cytochemistry and (**C**) ELISA using an anti-CPD antibody were assessed. DAPI was used to stain nuclear DNA. * Significantly different from untreated control cells (*p <* 0.05); and ^#^ significantly different from UVB-exposed cells (*p <* 0.05).

In our previous study, 20 μM DPHC protected HaCaT human keratinocyte cells from ultraviolet B radiation by attenuating oxidative stress [11]. Therefore, we used the same concentration of DPHC for all experiments in this study. Because UV light induces the formation of CPDs, which represent 70%–80% of the total UV-induced photoproducts, we determined the effect of DPHC on UVB-induced CPDs production by dot-blot analysis with a CPD antibody. Cells exposed to UVB (300 J/m^2^) contained more CPDs content than non-exposed cells, but DPHC significantly reduced the CPDs content (Figure 1A). We confirmed these results by immune-cytochemical analysis (Figure 1B) and ELISA analysis (Figure 1C). Taken together, these results indicate that DPHC protects HaCaT cells against UVB-induced CPDs.

### 2.2. Effect of DPHC against UVB-Reduced NER System

**Figure 2:**
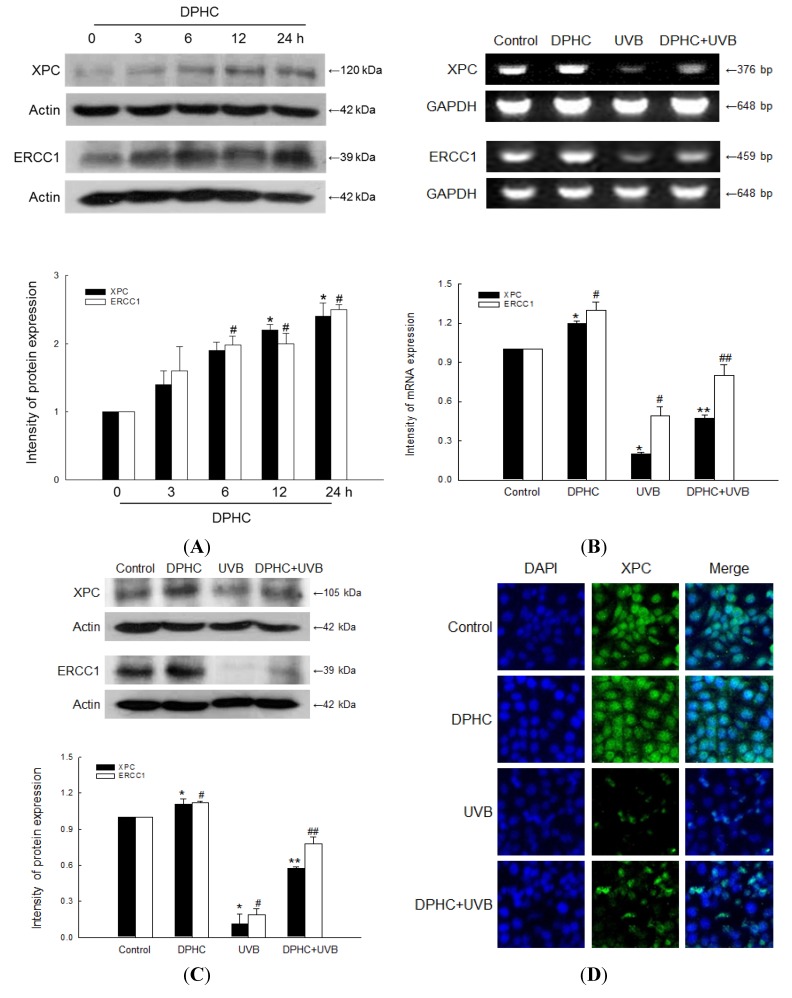

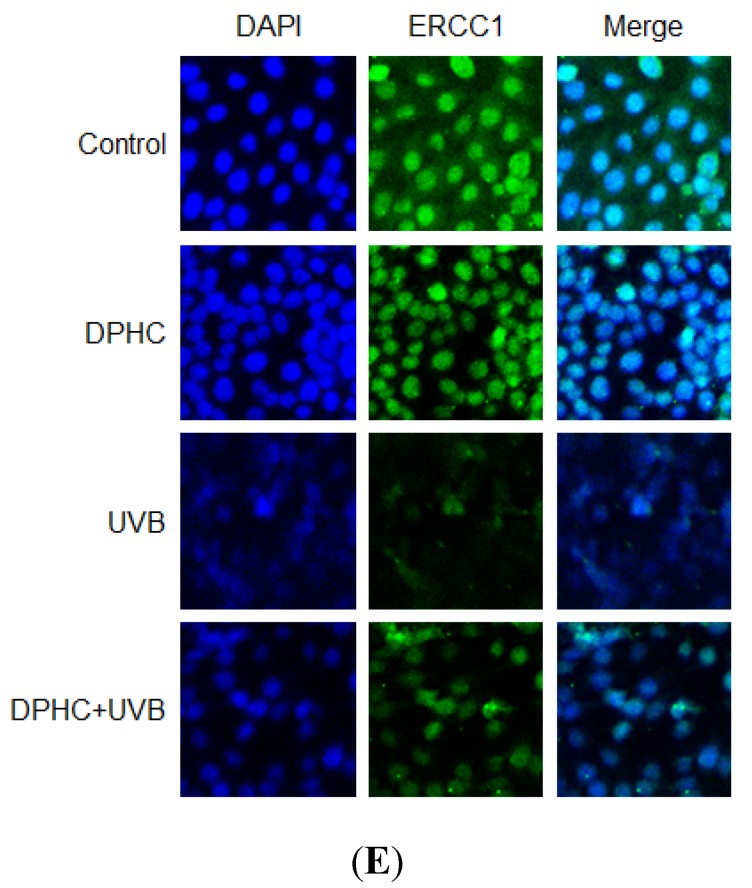
DPHC restores down-regulated xeroderma pigmentosum complementation group C (XPC) and excision repair cross-complementing 1 (ERCC1) expression following UVB irradiation in HaCaT human keratinocytes. (**A**) Cells were incubated in the presence of 20 μM DPHC for various times, and then Western-blot analysis of XPC and ERCC1 protein levels were performed. Actin was used as a loading control; *^,#^ significantly different from protein expression of XPC and ERCC1 in untreated control cells, respectively (*p <* 0.05). Cells were incubated in the presence of 20 μM DPHC for 1 h, and then irradiated with UVB; (**B**) RT-PCR analysis of XPC and ERCC1 mRNA levels were assessed 24 h after UVB irradiation. GAPDH was used as a loading control. *^,#^ significantly different from mRNA expression of XPC and ERCC1 in untreated control cells, respectively (*p <* 0.05); and **^,#^^#^ significantly different from mRNA expression of XPC and ERCC1 in UVB-exposed cells, respectively (*p <* 0.05); (**C**) Western-blot analysis of XPC and ERCC1 protein levels were performed 24 h after UVB irradiation. Actin was used as a loading control. *^,#^ significantly different from protein expression of XPC and ERCC1 in untreated control cells, respectively (*p <* 0.05); and **^,#^^#^ significantly different from protein expression of XPC and ERCC1 in UVB-exposed cells, respectively (*p <* 0.05); (**D**,**E**) Immune-cytochemical analysis of XPC and ERCC1 were performed 24 h after UVB irradiation. DAPI staining was used to determine the number of nuclei and to assess gross cell morphology.

XPC and ERCC1 are involved in the NER pathway which removes UV light-induced CPDs. We assessed whether DPHC affects protein expression of XPC and ERCC1 for various times in HaCaT cells. XPC and ERCC1 protein levels were induced at 12 h and at 6 h, respectively, in HaCaT cells (Figure 2A). XPC and ERCC1 mRNA levels were down-regulated in HaCaT cells following UVB irradiation; however, DPHC treatment partially restored the levels of both mRNAs (Figure 2B). Similarly, XPC and ERCC1 protein levels were down-regulated by UVB irradiation; again, DPHC treatment partially restored both protein levels, as determined by Western blot (Figure 2C) and immune-cytochemical analysis (Figure 2D,E).

### 2.3. Effect of DPHC on Sirtuin 1 (SIRT1) and Specificity Protein 1 (SP1) Levels in UVB-Exposed Cells

**Figure 3:**
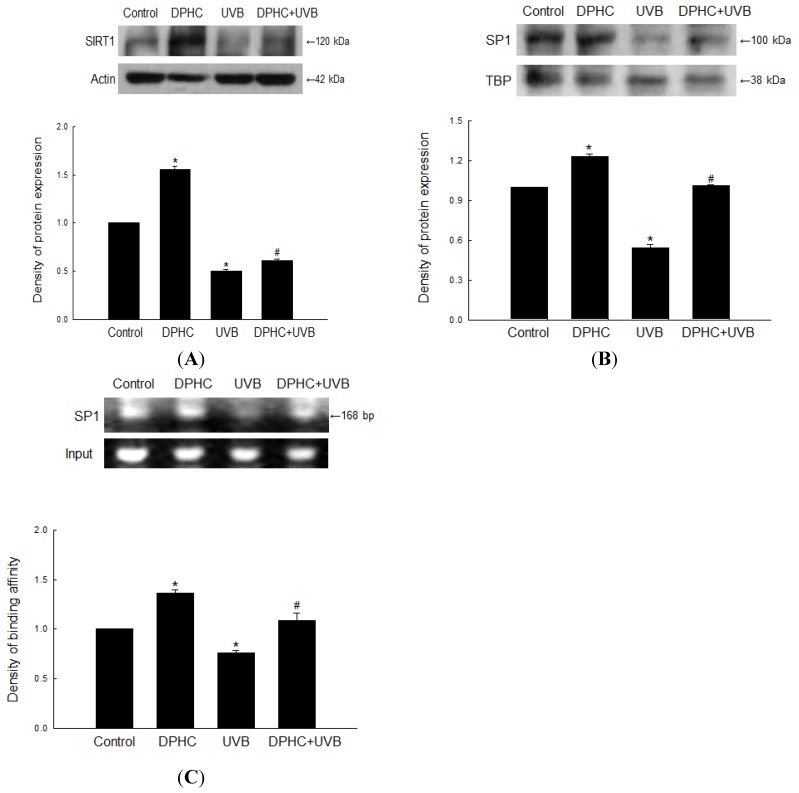
DPHC restores down-regulated expression of Sirtuin 1 (SIRT1) and Specificity Protein 1 (SP1) by UVB irradiation. Cells were incubated in the presence of DPHC for 1 h and then irradiated with UVB. (**A**) Western-blot analyses of SIRT1 protein expression were performed 24 h after UVB irradiation. Actin was used as a loading control; (**B**) Western blot analysis of SP1 protein level was assessed 24 h after UVB irradiation. TBP was used as a loading control; (**C**) ChIP analysis of SP1 binding to the XPC promoter were performed 24 h after UVB irradiation. * Significantly different from untreated control cells (*p <* 0.05); and ^#^ significantly different from UVB-exposed cells (*p <* 0.05).

It is demonstrated that loss of SIRT1 promotes activation of the oncogenic AKT pathway to mediate XPC down-regulation where mutation of the E2F site in the XPC promoter abolished the effect of SIRT1 inhibition and AKT activation, suggesting that inhibition of SIRT1 down-regulates XPC transcription by activating an AKT-dependent E2F pathway [17]. Western blot analysis revealed that exposure to UVB markedly decreased the expression of SIRT1, whereas DPHC partially restored the expression level (Figure 3A). We also performed Western blots to monitor expression of SP1, a transcription factor of XPC [18]. Exposure to UVB markedly reduced the expression of SP1, but DPHC partially restored SP1 expression level (Figure 3B). In addition, DPHC increased binding of SP1 to the XPC promoter, which was reduced in UVB-exposed cells (Figure 3C).

## 3. Discussion

The NER pathway is responsible for repairing bulky DNA lesions, including UV-induced photoproducts such as CPDs and 6-4 PPs [19]. NER damage recognition occurs via two sub-pathways, GGR and transcription-coupled repair. In GGR, lesions are recognized by DNA-binding protein complexes containing XPC and XPE, which recognize and bind to UV lesions, signaling the need for repair. The XPC protein plays an essential role in the initiation of GGR by recognizing DNA lesions and recruiting downstream factors. ERCC1 is also a highly conserved protein and an essential member of the NER pathway. The ERCC1/XPF heterodimer is a DNA structure-specific endonuclease that participates in NER by cutting DNA at junctions at which a single strand moves 5′ to 3′ away from a branch point with duplex DNA [20]. XPC and ERCC1 proteins are down-regulated by UVB [21,22]. Keratinocyte-specific deletion of the gene encoding ERCC1 in mice causes hypersensitivity to UV-induced skin cancer, demonstrating the critical role of this enzyme in repairing UV-induced DNA damage [23]. Our results revealed that DPHC elevated the expression of XPC and ERCC1 in both mRNA and protein levels suggesting the potential of DPHC to positively influence on repair system of UVB-induced CPDs, however more studies are needed to be carried out to elucidate their mechanisms. SIRT1 is an important regulator of cellular stress response and genomic integrity. Inhibition of SIRT1 impairs global genome NER by suppressing the transcription of XPC, suggesting that SIRT1 is required for XPC function [17,24]. In skin keratinocytes, SIRT1 expression is down-regulated by oxidative stress such as UVB or hydrogen peroxide [25]. In our system, DPHC partially restored the expression of SIRT1, which was reduced by UVB radiation. In addition to SIRT1, DPHC also up-regulated SP1, a transcription factor of XPC, and increased SP1 binding to the XPC promoter region [18].

Some natural compounds promote repair of UV-induced CPDs. For instance, silymarin protects cells from UVB-induced apoptotic cell death via induction of XPC gene expression [26]. In addition, ginsenoside Rb1 protects HaCaT cells from UV-induced apoptosis by inducing DNA repair systems such as XPC and ERCC1 [27]. Previous studies showed that oxidative stress in particular can inhibit NER capacity [28,29]. Recently, we reported that DPHC inhibits cell damage from UVB-induced oxidative stress in HaCaT cells both directly (via a ROS-scavenging effect) and indirectly (via induction of antioxidant enzymes) [11]. These results suggest that the antioxidant effects of DPHC can partially accelerate the removal of UVB-induced DNA lesions via the NER pathway.

## 4. Experimental Section

### 4.1. Reagents

Diphlorethohydroxycarmalol (DPHC) was provided by professor Nam Ho Lee of Jeju National University (Jeju, Korea). DPHC was dissolved in dimethyl sulfoxide (DMSO). The final concentration of DMSO in control or DPHC-treated samples did not exceed 0.05%. The CPD antibody (clone TDM-2) was purchased from Cosmo Bio Co., LTD (Tokyo, Japan). The CPD-HRP antibody (clone KTM53) was purchased from Kamiya Biomedical Company (Seattle, WA, USA). The actin antibody was purchased from Sigma-Aldrich (St. Louis, MO, USA). The XPC and TATA box bonding protein (TBP) antibodies were purchased from Abcam (Cambridge, MA, USA). The ERCC1, SP1, and sirtuin 1 (SIRT1) antibodies were purchased from Cell Signaling Technology (Beverly, MA, USA). All other chemicals and reagents were of analytical grade.

### 4.2. Cell Culture

HaCaT human keratinocytes (Amore Pacific, Yongin, Korea) were maintained at 37 °C in an incubator with a humidified atmosphere of 5% CO_2_/95% air. Cells were cultured in RPMI 1640 medium containing 10% heat-inactivated fetal bovine serum, 100 units/mL penicillin, 100 μg/mL streptomycin, and 0.25 μg/mL amphotericin B.

### 4.3. UVB Radiation

Cells were exposed to UVB light at a dose of 300 J/m^2^. A CL-1000M UV crosslinker (UVP, Upland, CA, USA) that delivered an UVB energy spectrum of 280–320 nm was used as the source. 

### 4.4. Dot Blot and ELISA Analysis

For detection of CPDs levels by using dot blot, genomic DNA was purified using the Wizard^®^ genomic DNA purification kit (Promega, Madison, WI, USA). DNA concentrations were measured using a Qubit fluorometer (Invitrogen, Camarillo, CA, USA) and the Quant-iT™ dsDNA HS assay kit (Invitrogen, Camarillo, CA, USA). Then, 500 ng of heat-denatured DNA was dotted onto a positively charged nitrocellulose membrane (GE Healthcare, Buckinghamshire, UK) pre-wetted in 6 × SSC. After blotting, the dots were rinsed twice with 100 μL of PBS. The membranes were blocked by incubating overnight at 4 °C in PBS containing 5% non-fat dry milk and 0.1% Tween 20, and then incubated for 1 h at 37 °C with an HRP-conjugated CPD antibody. After extensive washing with PBS containing 0.5% non-fat dry milk and 0.1% Tween 20, the membranes were incubated for 1 h at room temperature with a 1:2000 dilution of HRP-conjugated anti-mouse antibody in PBS-TPBS buffer containing 0.5% non-fat dry milk and 0.1% Tween 20. The blots were then washed extensively with the same buffer lacking the antibody, and peroxidase activity was determined using an enhanced chemiluminescence blotting detection system.

An ELISA was also used to determine the quantities of CPDs, as described by Mori *et al.* [30]. Briefly, 96-well cell culture plates pre-coated with 0.003% protamine sulfate were incubated with 10 ng of purified genomic DNA in PBS at 37 °C overnight. The CPD antibody (Cosmo Bio Co., Ltd., Tokyo, Japan) was used for detection, followed by incubation with biotin-conjugated F(ab′)_2_ fragment of anti-mouse IgG (Molecular Probes Inc., Eugene, OR, USA) and peroxidase-conjugated streptavidin (Invitrogen, Camarillo, CA, USA). The optical density of the substrate solution, consisting of 8 mg of *o*-phenylene diamine in 4 μL of 35% H_2_O_2_ and 20 mL citrate-phosphate buffer (pH 5.0), was measured at 492 nm using a VersaMax microplate reader (Molecular Devices, Sunnyvale, CA, USA).

### 4.5. Immune-Cytochemical Analysis

Cells plated on cover slips were fixed for 10 min with 100% ethanol, and then permeabilized for 20 min with 0.5% Triton X-100 in PBS. For detection of CPDs, cellular DNA was denatured by addition of 2 M HCl to each well for 30 min. To prevent non-specific binding, the cells were incubated with 20% FBS in PBS for 30 min at 37 °C. Subsequently, the cells were incubated for 30 min at 37 °C with the CPD antibody. Next, 200 μL of Alexa Fluor 594-conjugated F(ab′)2 fragment of anti-mouse IgG in PBS containing 5% FBS was added to each well, and the cells were incubated at 37 °C for 30 min with shaking. For detection of XPC and ERCC1, the fixed and permeabilized cells were treated with blocking solution (5% bovine serum albumin in PBS) for 1 h, and then incubated with XPC and ERCC1 antibodies diluted in 1% blocking solution for an additional 2 h. The immune-reacted primary antibodies were detected by incubation with a 1:500 dilution of FITC-conjugated secondary antibody (Jackson ImmunoResearch, West Grove, PA, USA) for 1 h. After washing with PBS, the stained cells were mounted onto microscope slides in mounting medium containing DAPI (Vector Laboratories, Burlingame, CA, USA). Images were collected on a Zeiss confocal microscope using the LSM 510 software.

### 4.6. Reverse Transcription-Polymerase Chain Reaction (RT-PCR)

Cells were seeded onto a culture plate at a density of 1 × 10^5^ cells/mL. Sixteen hours after plating, the cells were treated with 20 μM DPHC for 1 h, exposed to UVB (300 J/m^2^), and then incubated for an additional 24 h. Total RNA was isolated from cells using the easy-BLUE™ Total RNA extraction kit (iNtRON Biotechnology, Seongnam, Korea). The PCR conditions for XPC, ERCC1 and glyceraldehyde 3-phosphate dehydrogenase (GAPDH) were as follows: 35 cycles of 94 °C for 20 s, 60 °C for 10 s, and 72 °C for 30 s. The primer pairs (Bionics, Seoul, Korea) were as follows: XPC sense, 5′-CCC GCA AGT GCC GGG TTG AT-3′; XPC antisense, 5′-CGG GCT TTC CGA GCA CGG TT-3′; ERCC1 sense, 5′-GCA GGA GAG ACG CCC AAC CA-3′; ERCC1 antisense, 5′-CAG AGA CCG GGA GAC GAA GTC CT-3′; GAPDH sense, 5′-TCA AGT GGG GCG ATG CTG GC-3′; and GAPDH antisense, 5′-TGC CAG CCC CAG CGT CAA AG-3′. The amplified products were resolved by 1% agarose gel electrophoresis, stained with RedSafe™ nucleic acid staining solution (iNtRON Biotechnology, Seongnam, Korea), and photographed under UV light.

### 4.7. Western Blot Analysis

Harvested cells were lysed by incubation in 200 μL of PRO-PREP™ protein extraction solution (iNtRON Biotechnology, Seongnam, Korea) on ice for 10 min. The lysates were centrifuged at 13,000 rpm for 5 min, and the protein concentrations of the supernatants were determined. Aliquots of the lysates (30 μg of protein) were boiled for 5 min and electrophoresed on a 10% SDS-polyacrylamide gel. The resolved proteins were transferred onto nitrocellulose membranes, which were subsequently incubated with the appropriate primary antibodies, followed by incubation with secondary anti-IgG-HRP conjugates (Pierce, Rockford, IL, USA). Protein bands were detected using the Amersham™ ECL™ prime Western blotting detection reagent (GE Healthcare, Buckinghamshire, UK).

### 4.8. Chromatin Immune-Precipitation (ChIP)

The ChIP assay was performed using the SimpleChIP™ enzymatic chromatin IP kit (GE Healthcare, Buckinghamshire, UK), with slight modifications to the manufacturer’s protocol. Briefly, cells were cross-linked by the addition of 1% formaldehyde, and prepared chromatin was digested by incubation with micrococcal nuclease for 20 min at 37 °C. Normal rabbit IgG and rabbit monoclonal SP1 antibody were added to the chromatin digests, which were then incubated with constant rotation at 4 °C overnight. ChIP-grade protein G-agarose beads were added to capture the immune-precipitated complexes. The beads were then washed, and the immune-precipitates were eluted with ChIP elution buffer. Cross-links were reversed by incubating the eluate at 65 °C for 30 min, followed by incubation with proteinase K at 65 °C for 2 h. The immune-precipitated DNA fragments were purified on spin columns, and DNA recovered from the immune-precipitated complex was subjected to 45 cycles of PCR. The primers used were as follows: XPC promoter sense, 5′-GAG CCA TGT TGC TTG TCT GG-3′; XPC promoter antisense, 5′-TTT AGT GGC CAC GGG TAT GG-3′. The PCR products were separated on 2% agarose gels, and the DNA bands were visualized using RedSafe™ nucleic acid staining solution (iNtRON Biotechnology, Seongnam, Korea) on an Image Quant 350 (GE Healthcare, Buckinghamshire, UK).

### 4.9. Statistical Analysis

All measurements were performed in triplicate and all values were expressed as the mean ± standard error. Results were subjected to an analysis of variance (ANOVA), followed by Tukey’s *post-hoc* test to determine differences between the means. *p <* 0.05 was considered statistically significant.

## 5. Conclusions

In summary, our results demonstrated that DPHC can protect cells against UVB-induced DNA modifications by inducing the NER pathway of DNA repair. It remains to be determined whether topically applied DPHC can be used as a therapeutic agent to prevent UV-induced skin cancer and/or reduce the risk of developing skin cancer after chronic UVB exposure.
